# Estrogen receptors 1 and 2 genotypes and age at menarche in idiopathic scoliosis

**DOI:** 10.1186/1748-7161-10-S1-O8

**Published:** 2015-01-19

**Authors:** Piotr Janusz, Tomasz Kotwicki, Dariusz Czaprowski, Miroslaw Andrusiewicz, Malgorzata Kotwicka

**Affiliations:** 1 Spine Disorders Unit, Department of Pediatric Orthopedics and Traumatology, University of Medical Sciences, Poznan, Poland; 2 Department of Physiotherapy, Jozef Rusiecki University College, Olsztyn, Poland; 3 Department of Cell Biology University of Medical Sciences, Poznan, Poland

## Objectives

The age at menarche (AAM) is commonly in use in patients with IS as one of the maturity indicator suggesting deceleration of the growth velocity. The AAM was suggested to be related to predisposition and curve progression potential of IS. The late age at menarche was reported to be associated with higher prevalence of adolescent idiopathic scoliosis. The age at menarche is determined by both genetic and environmental factors as well as their interactions. Estrogen receptors 1 and 2 polymorphism were reported to be associated with AAM: in ESR1 XbaI and PvuII site polymorphism and in ESR2 AluI site polymorphism. The purpose of the study was to investigate associations of the ESR1 and ESR2 polymorphisms with AAM in IS patients.

## Material and methods

227 females with IS Caucasian females from Central Europe underwent clinical, radiological and genetic examinations. Four SNPs were selected XbaI (A/Grs9340799) and PvuII (C/T rs2234693) in ESR1and AluI (A/G rs4986938) and RasI (A/G rs1256049) in ESR2. Samples were analyzed with polymerase chain reaction followed by restriction fragments length polymorphism analysis (PCR-RFLP). The age of a menarche was established during personal interview with the patients and in case of children with their parents. The Cobb angle was measured.

## Results

All genotypes followed HWE. Mean AAM for patients was 154.7±14 months (12.9±1.2 years). The earliest AAM was 123 and latest 185 months. There was no statistically significant difference between AAM mean values in each genotype. Patients divided according to Cobb into mild (<30º), moderate (30º-49º) or severe (>49º) IS revealed tendency to delay AAM: 152.8±14.4; 154.3±15.7 and 157.9±14.0 months, respectively. There was statistical significant difference between patients with mild <30º and severe >49º IS, p=0.0433

## Conclusions

In IS patients estrogen receptors polymorphisms did not show association with the AAM. Patients with severe IS form revealed delayed AAM than patients with mild IS form.

